# Ahead of the Curve: Leveraging Antecedents of Corporate Entrepreneurship to Pull Off Competitive Advantage

**DOI:** 10.3389/fpsyg.2020.531886

**Published:** 2020-10-26

**Authors:** Asif Mahmood, Ahmad Arslan

**Affiliations:** ^1^Department of Business Studies, Namal Institute, Mianwali, Pakistan; ^2^Institute of Business and Management, University of Engineering and Technology, Lahore, Pakistan

**Keywords:** corporate entrepreneurship, information and communication technologies, innovation, absorptive capacity, chief executive officer’s temporal leadership, competitive advantage

## Abstract

Entrepreneurship is a highly dynamic and important endeavor that spills over to economic, technological, and social canvas of a society in this rapidly changing globalized economy. The purpose of the present quantitative study is to investigate the associations among information and communication technologies, innovation, absorptive capacity, CEO’s temporal leadership, and competitive advantage by considering corporate entrepreneurship as a mediator. These factors have been incorporated because they play a predominant role to vie in a competitive environment for entrepreneurial success and economic growth. We used the response of 460 organizations, acquired on a Likert scale, to examine how antecedents of corporate entrepreneurship contribute toward competitive advantage. Structural equation modeling was employed to analyze the measurement and structural relationships including the mediation effects of corporate entrepreneurship. All the relationships with corporate entrepreneurship were found significant except the direct effect of absorptive capacity on competitive advantage. Hence, the results established corporate entrepreneurship as a mediator to predict competitive advantage partially by information and communication technologies (ICT) use, innovation, and temporal leadership. The findings also reveal that absorptive capacity reaps an entire competitive advantage only through corporate entrepreneurship. Practically, the study would be invaluable for organizations, entrepreneurs, and managers to capture a lot of opportunities in effectively managing scarce resources.

## Introduction

The new economic and business setting caused by complex technological advances and an uncertain environment calls for faster and innovative response strategies to maintain competitive advantage. With the inability to perceive this departure, many projects failed, and consequently, organizations were unsuccessful to achieve their planned goals (Yunis et al., 2018). Recently, a study by Klynveld Peat Marwick Goerdeler (KPMG, a professional service company and one of the Big Four auditors) has examined that 70% of businesses suffered loss in their projects and 50% failed to attain their intended goals (Amankwah-Amoah, 2016). Gartner (2012) stated that 55–75% of projects of enterprise resource planning (ERP) and more than 70% of information system projects did not achieve their businesses goals. Moreover, 74.1% of these suffered loss due to excessive cost, while 50% are not realizing benefits (Jacobs, 2012). Gartner also reported that only 30% of projects of information system attain business objectives (Saran, 2012). The aforementioned projects’ failures could be attributed to numerous problems such as the lack of entrepreneurial activities and poor competitive strategy positioning of IT firms (Carlton, 2014).

Corporate entrepreneurship (CE) is a riposte for the survival and competitive success of business entities in this current situation (Bojica and Fuentes, 2012). Entrepreneurial organizations hinge on specific attitudes and behaviors. This organizational entrepreneurial behavior is bespoken by its transfiguration into a superior entity, emerging out of pattern of resource deployment. Both the frequency and success of endeavors revolve around the configuration of strategic assets such as information and communication technologies (ICT), innovation, absorptive capacity, and temporal leadership. Therefore, it is necessary for the academic community to study the psychology of entrepreneurship in order to discover new horizons (Baum et al., 2007). However, being a novel research area, the psychology of entrepreneurship is yet to be explored in economic, social, personal, and societal contexts (Gorgievski and Stephan, 2016). Previous studies (for example, Kuratko and Audretsch, 2013) have examined how corporate entrepreneurship relates with firms’ resources such as innovation and ICT. However, there is still a need for integrated studies in today’s knowledge-based globalized economy to analyze the impact of ICT and innovation on competitive advantage while considering the mediating role of corporate entrepreneurship (Yunis et al., 2018). Although corporate entrepreneurship has immense prospect to establish competitive advantage, the configuration of individual antecedents of CE poses considerable challenges (Mostafiz, 2020). There are many studies that accentuate CE (for example, Chen S. et al., 2015; Burgers and Covin, 2016; Mazzei et al., 2017), but how it ties in with ICT, innovation, absorptive capacity, temporal leadership, and competitive advantage remains unexplored. Therefore, the role of these strategic assets in promoting corporate entrepreneurial activities warrants additional research. Conclusively, it can be enounced that the mere focus on CE is inescapable but not enough to outclass the competition. Thus, in this study, we draw on this frame of reference to develop and examine the forenamed links. In order to make a doable study, we have identified four types of inquiries into corporate entrepreneurship leading toward competitive advantage: ICT use, innovation, absorptive capacity, and CEO’s temporal leadership. The subsequent illustration of corporate entrepreneurship, its antecedents, and consequences would help better understand the psychology of the whole entrepreneurial process.

Corporate entrepreneurship is the ability of an organization to explore and exploit profitable opportunities without being inhibited by limitations of resources, rules, and regulations, as well as managerial decisions (Otache and Mahmood, 2015). It may also be viewed as a set of firms’ activities that involve innovation, corporate venturing, and strategic renewal acting as a main driving force in achieving competitive advantage by entering into the external environment (Zahra, 1996). So, the entrepreneurial activities of the organizations (corporate entrepreneurship) can be regarded as corporate venturing, risk-taking, innovation, strategic renewal, and proactiveness. There are different elements that drive corporate entrepreneurship, for example, cultural diversity, organizational structure, etc. (Covin et al., 2006). Likewise, the entrepreneurial spirit is swayed by different factors such as ICT use, innovation, absorptive capacity, and CEO’s temporal leadership (Chen S. et al., 2015; Burgers and Covin, 2016; Mazzei et al., 2017).

ICT use can be defined as a “diverse set of technological tools and resources used to create, disseminate, store, and manage information” (Blurton, 2005, p. 1). The effective utilization of ICT resources provides new opportunities for developing novel products, business models, and services. The fast changing environment of business has increased the dependence on ICT that, in turn, has pushed it toward innovative activities for obtaining higher efficiency and attaining competitive advantage in a dynamic market (Igun, 2014). Therefore, innovations are also very important for the growth of a company and competing with other organizations in the current dynamic and competitive environment. It is a process that increases the firms’ value web and chain by way of services, new products, procedures of work, commercialization system, and solutions (McFadzean et al., 2005). Innovation also focuses on those activities that show change into the present business patterns, and develops new business ventures that lead toward new product formation by creating new markets (Kuratko and Audretsch, 2013; Ramos-González et al., 2017). A profound study of corporate entrepreneurship and its role can integrate ICT and innovation into the firm’s beneficial resources and strategies for achieving a higher level of competitive advantage (Yunis et al., 2018). ICT and innovation are generally strategic resources of an organization which can develop the firm’s activities through entrepreneur behavior and ability (Yunis et al., 2017).

One of the other entrepreneurial constituents is absorptive capacity which can be defined as “the organization’s relative ability to develop a set of organizational routines and strategic processes through which it acquires, assimilates, transforms and exploits knowledge acquired from outside the organization in order to create value” (Jiménez-Barrionuevo et al., 2019, p. 3037). In other words, it is the ability through which organizations can develop, learn, integrate, and apply new knowledge (Najafi Tavani et al., 2013). It not only develops available knowledge of organizations but also encourages for the creation of innovative knowledge activities that lead toward entrepreneurial success (Bojica and Fuentes, 2012). Firms which continuously invest in adapting and taking advantage of new external knowledge are most likely to capitalize on an ever fluctuating competitive environment and generate new innovative products. Firms should develop this capacity if they wish to adapt to changes in an increasingly competitive and changing environment (Jiménez-Barrionuevo et al., 2019).

Similarly, due to fast changes in customer likening, advancement in technologies, and competition, firms are now forced to think about time. This time issue has brought organizations to the frontline for research in strategic management (Bridoux et al., 2013). According to the dynamic capability theory, timely approachability to the market dynamics and fast products innovation decides the organizations’ success and helps them gain competitive advantage. Therefore, by proficiently allocating temporal resources, firms can lead toward strategic initiatives to innovate. It also must ensure that top management teams dedicate their important time to supervise the corporate entrepreneurial activities (Shimizu, 2012). Temporal leadership is a set of leader’s behavior related to the temporal traits of team tasks that comprise three activities: *allocation of temporal resources*, *scheduling*, and *temporal synchronization* (Mohammed and Nadkarni, 2011). The allocation of temporal resources involves the distribution of time in the activities of the team efficiently and effectively, specifically when time pressure is at the extreme (Mohammed and Nadkarni, 2011). Scheduling is a specific timeline for completing the team activities, whereas temporal synchronization involves coordination and temporally sequencing the team members’ activities and addresses the question of how to complete the task. The leaders give priority to team goals, allocation of time for subtasks, and form time built-in blocks for unpredicted contingency gaps, for example, configuration of team members and development of coordination among them on a specific time (Maruping et al., 2015). They also make a clear framework to ensure that every member of the team completes his or her task timely, and continually modify this framework while accommodating deviations, delays, and gaps (Maruping et al., 2015).

Considering the foregoing discussion, the interplay of CE with the most promising antecedents and its dénouement in the form of competitive advantage is the focus of current research. The study, designed on this premise, would help managers, entrepreneurs, innovation adopters, and technology suppliers to capitalize on dynamic capabilities and value creation resources (corporate entrepreneurship, ICT, innovation, absorptive capacity, and temporal leadership). These resources, once transformed into competitive advantage, would help face the global challenges.

## Literature Review and Hypotheses Development

### Information and Communication Technologies Use and Corporate Entrepreneurship

In the contemporary competitive world, entrepreneurs operate their business in a technology-rich environment. Entrepreneurs must also perform their activities earnestly while using tools of computing, online communication, and cooperation (van Laar et al., 2017). ICT use improves productivity by contributing not only toward effectiveness in operations and inventory management but also toward the integration of activities (Igun, 2014; Liao et al., 2015). The adoption and effective use of ICT affect both operational efficiency and economic growth in public and private institutions. Therefore, it has become inevitable for organizations to adopt new technologies like ICT to survive in a rapidly changing business environment (Shah Alam et al., 2011).

However, investment in ICT should not be made in isolation, but it must be aligned with goals, missions, strategies, and directions of the organization. It must also be adopted according to users’ requirements, and ICT jobs must be well determined (Pagano and Brügge, 2013). Tang et al. (2015) have examined that corporate entrepreneurship is influenced by IT skills in myriad ways, for example, revitalizing and revamping the structure of business, supporting functions for making and sharing information, enhancing the system of communication and their outcome of interrelated parts, etc. While examining the role of ICT and entrepreneurship development in Iran, six advantages were found which are as follows: improvement of infrastructure services, motivation promotion, improvement in business performance, organizational factor, technology, and information factor (Hosseini et al., 2014). In a nutshell, ICT not only supports corporate activities but also provides basis for implementation of new network, firm practices, human capital training, and development of labor polices and spillovers the effects of technology and knowledge (Venturi, 2015). Thus,

H_1_: ICT use has a significant impact on corporate entrepreneurship.

### Innovation and Corporate Entrepreneurship

Innovative organizations preemptively use innovation strategies for making business models, services, and new products and, hence, build a strong relationship between innovation and entrepreneurship to outperform in competitive markets. Due to the fast growing progress in technology and science, product innovation has become an overriding concern for those firms who are struggling to achieve competitive advantage (Chen S. et al., 2015; Chen Y. et al., 2015). For firms that adopt corporate entrepreneurship in their businesses, creation of new products becomes necessary for them (Kuratko et al., 2015). Innovation-based corporate entrepreneurship is a development that emphasizes and clarifies the relationship between research areas of corporate entrepreneurship and innovation (Salamzadeh and Kirby, 2017). Innovation and entrepreneurship are positively related to each other to help an organization to be more successful and expansive (de Jong, 2013; Jarrar and Smith, 2014; Urban and Wood, 2015). Technological innovation can play a significant role to achieve higher level economic benefits by facilitating the production of new goods and services if it is well arranged and supported. Research on innovation at the organization level shows the significance of corporate entrepreneurship while exploiting the innovative opportunities (Szirmai et al., 2013). It can also be said that technological innovation adoption alone is not enough to sustain competitive advantage, but the benefits can be achieved through more systematic and complex ways (Martín-Rojas et al., 2017). In modern businesses, fusion of innovation and entrepreneurship is a high-level strategy for achieving success (Kuratko et al., 2014). Considering the abovementioned discussion, we may propose the following hypothesis:

H_2_: Innovation has a significant impact on corporate entrepreneurship.

### Absorptive Capacity and Corporate Entrepreneurship

Firms with absorptive capacity increase their performance through access of external knowledge and show their willingness to reciprocate toward the external environment by innovative ways (García-Sánchez et al., 2018). Absorptive capacity also plays a significant role to determine the range of knowledge flows (Hurmelinna-Laukkanen et al., 2012). External knowledge exploitation supports firms to increase their knowledge base and identify new opportunities that are present in the market, as well as sponsor the new products and technologies to manage their resources effectively (Forés and Camisón, 2016). Knowledge absorptive capacity can be constantly utilized to acquire and digest external knowledge. Therefore, it becomes important for an organization to identify opportunities in the market by using this new or external knowledge to get innovation (Xie et al., 2018). Organizations from external sources gain and exploit knowledge to improve their resources (Ali et al., 2016). According to the knowledge base theory, absorptive capacity significantly increases the capacity of an organization to recognize and find out new opportunities by reducing cognitive inflexibility and developing new abilities among top executives (Espejo and Dominici, 2017). Absorptive capacity has a direct impact on factors that promote corporate entrepreneurial system (Belderbos et al., 2016). Researchers have found significant direct and indirect relationships between absorptive capacity and a firm’s entrepreneurial performance (Bharati et al., 2014). Realized absorptive capacity brings new ideas within the firm, increases the capability to recognize these novel ideas, creates strength, and ultimately, develops the ability to understand opportunities (Cepeda-Carrion et al., 2012). Some studies also considered the issue that absorptive capacity is strategically important for creating new opportunities for business by encouraging corporate entrepreneurship and enhancing firm performance (Martín-Rojas et al., 2011).

Considering the abovementioned discussion, we may propose the following hypothesis:

H_3_: Absorptive capacity has a significant impact on corporate entrepreneurship.

### CEO’s Temporal Leadership and Corporate Entrepreneurship

Temporal leadership, managed by leaders to meet deadlines, is an important factor for entrepreneurship because it acts as a coordinator between work, various time frames, and member contributions (Mohammed and Alipour, 2014). CEOs’ temporal leadership and their behavior related to temporal aspects of higher management team affairs are the important mechanisms of CEOs’ pacing style and time urgency in shaping strategic activities of organizations (Mohammed and Nadkarni, 2011). It represents arrangements and development of activities, allocation of temporal resources, and synchronization of activities for the completion of tasks. The clarification of schedules and the allocation of temporal resources effectively reduce the ambiguity of tasks completion, disagreements, meeting deadlines, and how teams perform activities of tasks. These also help understand how team members spend time on every task to meet the targets (Standifer et al., 2015). Chalking out coherent schedules, making long-term objectives, and setting temporal milestones and subtasks not only help the top management team in providing clear directions to the firm’s members for corporate entrepreneurship activities inside the organizations but also facilitate in performing corporate entrepreneurial activities across the firm within time frames. So, the coherent scheduling of strategic actions outside the firm ensures a clear and combined plan of activities within the top management team members for framing and applying corporate entrepreneurial activities. Therefore, these schedules not only assist top management teams to watch the progress of every activity but also support timely completion of initiatives of corporate entrepreneurship (Chen and Nadkarni, 2017). However, toward the timely completion of goals, the leader and follower must be properly sequenced to energetically regulate the individual work activities, which is not possible without a strong temporal leadership. This discrepancy between the follower temporal behavior and leader’s ideal temporal prototype will lead toward failure of coordination (Alipour et al., 2017). Therefore, since the temporal leadership behavior is employed by the team leader, it must be ensured that all team members agree on tasks policies and must follow these strategies and allocate temporal resources efficiently toward the tasks (Mohammed and Nadkarni, 2011). Furthermore, temporal leadership helps team members take advantage of optimistic effects of intermediate levels of time pressure because entrepreneurs see time pressure an aspect of motivation. Therefore, temporal leadership supports entrepreneurs to take positive benefit of time pressure who make their plans and activities according to time constraints (Maruping et al., 2015). In view of the foregoing discussion, we may propose the following hypothesis:

H_4_: CEO’s temporal leadership has a significant impact on corporate entrepreneurship.

### Corporate Entrepreneurship and Competitive Advantage

The success of entrepreneurship is associated with unique knowledge, skills of executives, and experience of entrepreneurs (Staniewski, 2016). Experience, acquired from any type of entrepreneurship, increases the probability of undertaking corporate entrepreneurship (Urbano and Turró, 2013). The individual knowledge and experience acquired from prior entrepreneurial activities also influence further intentions to enhance growth (Miralles et al., 2016). Therefore, it can be enunciated that corporate entrepreneurship is not only an activity of a firm’s capabilities but also it is about how these capabilities are beneficial to achieve the desired result (Stevens et al., 2015). Specifically, the relationship between corporate entrepreneurship and competitive advantage is determined by non-financial and financial measures of latent variables. The non-financial measures comprise satisfaction and global success of business owners and managers (Daryani and Karimi, 2017; Prange and Pinho, 2017), whereas financial measures consist of revenues, return on capital, profit, return on assets, and return on equity among others. Hence, corporate entrepreneurship is a strong promoter of growth for new and existing businesses (Chen S. et al., 2015; Chen Y. et al., 2015). Taking into account these arguments, we may propose the following hypothesis:

H_5_: Corporate entrepreneurship has a significant impact on competitive advantage.

### ICT Use and Competitive Advantage

Information and communication technologies is the most significant element for economic development, and its extraordinary functions have brought fundamental changes for the development of research and education. Corporate entrepreneurship enabled by innovation and ICT aligns a firm’s strategies and resources because it constitutes dimensions that are vital for an organization to attain competitive advantage (Kuratko and Audretsch, 2013). Organizing resources of ICT toward increasing firm performance and attaining competitive advantage needs a firm culture, which in turn can support in finding and assessing new opportunities and making use of these new avenues (Agarwal and Brem, 2015). Therefore, ICT use is the core factor for entrepreneurial development that contributes toward new job opportunities in e-markets, and also facilitates in selling the merchandise in cyberspace (Haghighi et al., 2018). Thus, it can be articulated that the role of information and communications technology is not only as a tool to increase efficiency of a firm’s internal processes but also as a source to attain competitive advantage (Lusch and Nambisan, 2015). In this way, ICT, playing a critical role as the alpha and omega of competitive advantage, leads toward lower cost and better services (Andersen, 2015; Cohen and Olsen, 2015). Consequentially, the effective use of ICT adoption contributes toward competitive advantage and a successful organization (Manochehri et al., 2012). Hence, we propose the following hypothesis:

H_6_: ICT has a significant impact on competitive advantage.

### Innovation and Competitive Advantage

Due to multitudinous changes in the global world, corporate entrepreneurship starts new activities for organizations, follows new innovation processes, and takes interest in departing from the daily unchanging process for exploring, creating, and chasing new profitable opportunities (García-Morales et al., 2014). In order to achieve success in this competitive environment, product development is indispensable, and the literature suggests that it is a component of corporate entrepreneurial movements (Kuratko and Audretsch, 2013). Today, undeterred by the changing economies, business innovation in products and services plays an important role. In this age of technology and competitive environment, unique and dynamic business innovations are very essential for the growth of business and in vying for the market share (Malaquias et al., 2016). As innovation can transform ICT resources, a firm’s practices, and explicit and tacit knowledge into beneficial capabilities, therefore, competitive advantage can be achieved through innovation (Agarwal and Brem, 2015). However, in order to achieve a higher level of competitive advantage and opportunities, ICT resources and innovations must be well organized (Agarwal and Brem, 2015). Thus, we propose the following hypothesis:

H_7_: Innovation has a significant impact on competitive advantage.

### Absorptive Capacity and Competitive Advantage

Absorptive capacity can integrate internal and external knowledge for the firms to be employed to develop new products and services. In order to increase absorptive capacity, global enterprises make use of digital platforms to combine internal and external knowledge (Audretsch et al., 2014). An organization that constantly invests in integrating and exploiting new external knowledge gains advantages in emerging markets and a rapidly changing environment by developing innovative products (Rangus and Slavec, 2017). Absorptive capacity and corporate entrepreneurship are considered the key elements of the dynamic capabilities of an organization. The dynamic capability of a firm refers to how a firm utilizes its internal and external resources and deploy, redeploy, and reconfigure them for gaining a competitive advantage (Rehman et al., 2020). In general, a firm’s ability to acquire, reconfigure, and integrate knowledge and understand innovative technologies bolster its competitive advantage (Chang et al., 2014). As a matter of fact, firms obtain knowledge through potential absorptive capacity and exploit it to reconfigure for their benefit through realized absorptive capacity (Ben-Oz and Greve, 2015; Leal-Rodríguez et al., 2015; Ali and Park, 2016). In a nutshell, firms accompanied by their proactive absorptive capacity hone their expertise to reciprocate to the dynamic environment, and provide the best opportunities to enhance competitive advantage by framing the entrepreneurial strategy (Bojica and Fuentes, 2012). Taking the preceding arguments into account, we propose the following hypothesis:

H_8_: Absorptive capacity has a significant impact on competitive advantage.

### CEO’s Temporal Leadership and Competitive Advantage

The behavior of temporal leadership determines how teams respond effectively to time pressure. It supports members of the team in managing time, planning work, and attaining competitive advantage (Mohammed and Nadkarni, 2011; Maruping et al., 2015). So, the behavior of temporal leadership helps team members plan their tasks and manage time in order to attain competitive advantage (Mohammed and Nadkarni, 2011). Temporal leadership has a positive relationship with entrepreneurship and also influences the competitive advantage (Mohammed and Nadkarni, 2011; Maruping et al., 2015). Moreover, how CEOs think and feel about time may have a colossal predominance in crafting their firm’s strategies (Chen and Nadkarni, 2017). Therefore, we are of the view that:

H_9_: CEO’s temporal leadership has a significant impact on competitive advantage.
H_10_: Corporate Entrepreneurship as a Mediator.

Since the industry is changing from traditional to economy-based as part of globalization, corporate entrepreneurship plays an important role in attaining a higher level of competitive advantage (Kuratko and Audretsch, 2013). The competitive and globalized environment poses myriad challenges. Despite these challenges, a lot of opportunities and competitive advantages can be achieved by organizations using ICT, innovative resources, and dynamic capabilities which, in turn, depend on the behavior of entrepreneurs (Yunis et al., 2018). ICT and innovation play an important role to cultivate corporate entrepreneurship and, consequently, increase organizational performance to the higher level by using the organization’s resources and well-shaped strategies (Mortara et al., 2011). Corporate entrepreneurship is a situation embodied in organizational capabilities to effectuate competitive advantage (Stevens et al., 2015). In a competitive business environment, absorptive capacity and corporate entrepreneurship are considered a direct source of high performance for a firm. IT infrastructure flexibility facilitates a firm with exchanging knowledge, novelty in products, and new business venturing, which then helps in sustaining the competitive advantage (Jiménez-Barrionuevo et al., 2019; Martin-Rojas et al., 2019). Absorptive capacity enables firms to use the transmission of knowledge to pursue corporate entrepreneurship and also helps in meeting the looming challenges to corporate entrepreneurship (Jiménez-Barrionuevo et al., 2019). Organizations that exhibit corporate entrepreneurship are usually perceived as dynamic, flexible entities that prepare themselves to take benefit of new business opportunities (de Jong, 2013; Busenitz et al., 2014). Corporate entrepreneurship and accompanying activities are quite valuable for the firms’ growth, productivity, and profitability, for they partake substantially in imparting novel ideas within organizations (Chen Y. et al., 2015). Therefore, CE plays a significant role for attaining the highest level of competitive advantage, productivity, and benefits of competitiveness (Agarwal and Brem, 2015). As a matter of course, CE is still a fitting factor that firms can resort to in order to attain competitive advantage and gain financial control in a competitive environment (Sarooghi et al., 2015). It also contributes to the ongoing activities of a business such as risk-taking, innovation, self-renewal, new business venturing, and proactivity (Chen Y. et al., 2015; Burgers and Covin, 2016; Mazzei et al., 2017). Therefore, based upon the cogent lines of argumentation, we assert the following:

H_10__–__A_: Corporate entrepreneurship mediates the relationship between ICT use and competitive advantage.
H_10__–__B_: Corporate entrepreneurship mediates the relationship between innovation and competitive advantage.
H_10__–__C_: Corporate entrepreneurship mediates the relationship between absorptive capacity, CEO’s temporal leadership, and competitive advantage.
H_10__–__D_: Corporate entrepreneurship mediates the relationship between ICT use, innovation, absorptive capacity, CEO’s temporal leadership, and competitive advantage.

**FIGURE 1:**
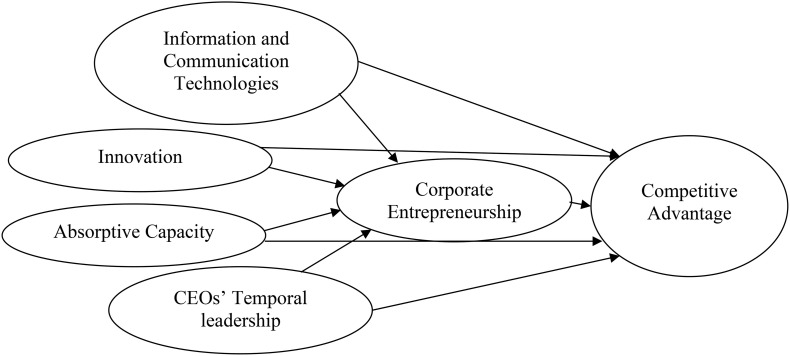
Research model.

Based upon the foregoing hypotheses, the research model is described in Figure 1.

## Methods

### Participants and Procedure

The target population for this study comprises both middle- and upper-level employees working in Lahore-based IT sector of Pakistan. Being deductive in nature, the research employs a quantitative approach including a well-structured questionnaire. A convenient sampling process was used for collection of data from 460 firms through emails and direct meetings. The representative employees had knowledge about the use of ICT and orientation of innovation and were well-experienced about how to take advantage of absorptive capacity through entrepreneurial behavior in their firms. Although 600 questionnaires were distributed to the IT firms, only 495 of them were received and only 460 were found complete in all respects. Thus, the eventual response rate was computed to be 76.66%.

The sample size is consistent with the recommendations by Pallant (2005) and Kline (2010) to execute structural equation modeling (SEM)-based analyses in AMOS software. The characteristics of the study sample are reported in Table 1.

**TABLE 1:** Demographic characteristics of the respondents.

	**Frequency**	**Percent**	**Cumulative percent**
**Gender**			
Male	415	90.22	90.22
Female	45	9.6	100
**Age**			
21–30 years	55	11.96	11.96
31–40 years	286	62.17	74.13
41–50 years	113	24.57	98.70
>50 years	6	1.30	100
**Experience**			
Less than and equal to 10 years	90	19.57	19.57
11–20 years	332	72.17	91.74
20 years and above	38	8.26	100
**Designation**			
Middle management	430	93.48	93.48
Upper management	30	6.52	100

It can be observed from the gender-wise frequency analysis of the data that 90.4% of the sample comprised male employees, while 9.6% of the respondents were female. Hence, the dominant majority of the respondents were male in this data. As per the age frequency, 55 of the respondents, i.e., 11.96% of the total sample, belonged to the age group of 21–30 years, while in the age bracket of 31–40 years, there were 286 respondents equivalent to 62.17%. In the age cohort of 41–50 years, the number of respondents was 113, which is 24.57% of the total respondents, whereas 6 respondents belonged to the age group greater than 51 years (1.3%). The survey also collected data about work experience of the respondents. There were 90 respondents who had work experience of 1 to 10 years (19.57%), and there were 332 of the respondents who possessed work experience of 11–20 years, i.e., 72.17%. However, in the other experience category of 20 years and above, there were 38 respondents making up 8.26% of the total sample size. The designation of the respondents is another aspect of demographics which indicates that a total of 430 respondents belong to middle management making up 93.5% of the total sample. The remaining 30 respondents were part of the upper management (6.52%).

### Measures

This study is a correlational design to examine the relationships among ICT use, innovation, temporal leadership, absorptive capacity, and corporate entrepreneurship to explore the potential causal impact of each of these factors on competitive advantage. As this research is deductive and quantitative in nature, it utilizes well-structured measurement scales made up of items denoting the respondents’ thoughts and opinions about ICT use, innovation, temporal leadership, absorptive capacity, corporate entrepreneurship, and competitive advantage in their businesses. All items computing these attitudinal variables used the five-point Likert scale response format (1 for strongly disagree, 5 for strongly agree). Information and communication technology (ICT use), used as the independent variable, was measured by a four-item scale developed by Davis (1989), and further validated by Rogers (1995) and Agarwal and Prasad (1998) with sufficient reliability value (Cronbach’s alpha = 0.84). An 11-item measuring scale, based on Gatignon et al. (2002), was used to measure another independent variable “innovation.” The reported Cronbach’s alpha value is 0.91. Similarly, an 11-item scale (Jiménez-Barrionuevo et al., 2019) was applied to measure another independent variable “absorptive capacity.” In this study, the internal consistency value was observed to be 0.93. Likewise, a seven-item scale by Mohammed and Nadkarni (2011) was employed to measure the independent variable “temporal leadership.” The study stated a reliability value of 0.87. In order to measure the mediator “corporate entrepreneurship,” a six-item scale, with Cronbach’s alpha value of 0.88, was utilized based on Zahra (1996). Finally, to evaluate the dependent variable competitive advantage, a seven-item scale, based on McDougall et al. (1994), was used. The internal consistency value was noted to be 0.89.

## Data Analysis and Results

### Research Design

Structural equation modeling has been used with the help of AMOS 24 for testing the proposed hypotheses empirically. SEM has two elements: the first is confirmatory factor analysis (CFA), which is used to measure the validity of a model comprising unobserved and observed variables, and second component is path analysis that is used to fit the structural model with the latent variables (Kline, 2010). In the first assessment, there is checking of the validity of indicators, whereas the second assessment specifies the process in which a certain latent variable directly or indirectly becomes a cause to change in other latent variable (Byrne, 2001). This two-step method guarantees that only the constructs with appropriate measures might be used in the structural model. Furthermore, measurement and structural models were evaluated through three fit measures, i.e., goodness of fit index (GFI), relative chi-square ratio over degree of freedom (χ^2^/*DF*) and root mean square error approximation (RMSEA).

Due to the cross-sectional nature of the study, potential method biases caused by common method variance (CMV) may be present in the data collected (Spector 1994; Podsakoff et al., 2003). Therefore, it has to be checked to trace the degree of biasness. Statistical techniques were used to restrict CMV. First, a *post hoc* Harman’s single factor test (Chang et al., 2010) was carried out with unrotated factor. The test reported 23% variance explained by the combined factor, which is lower than the recommended value of 50% (Podsakoff et al., 2003). Hence, it supported the fact that common method bias was not a considerable concern in this study. Furthermore, the accumulated variance explained by individual factors was 65%, which additionally vindicated the claim. Apart from this test, confirmatory factor analysis of the single factor was also conducted to trace the method biases, in case the data fits the hypothesized model (Malhotra et al., 2006). The poor fit of the data for the single factor substantiates the absence of CMV [χ^2^/*DF* = 9.663, GFI = 0.281, AGFI = 0.229, normed fit index (NFI) = 0.262, incremental fit index (IFI) = 0.284, TLI = 0.247, RMR = 0.103, and RMSEA = 0.150]. We also employed the common latent factor (CLF) test. The standardized regression weights of the model with and without CLF were juxtaposed, and the deviations less than 25% gave credence to non-existence of CMV (Williams et al., 1989).

### Descriptive Statistics

We calculated means, skewness, and kurtosis for all the six latent constructs. The descriptive statistics given below in Table 2 indicate positive behavior of the items. The standard deviation (SD) has a range of values from 0.49584 to 0.76434; the mean value has also a range of values from 3.5631 to 4.3407, which is greater than the midpoint (2.5). Moreover, that data is distributed normally based on the values of skewness and kurtosis. The values of skewness and kurtosis were found within the range of normality, i.e., −1.0 to +1.0 for skewness, and for kurtosis less than 10 (Kline, 2010). Furthermore, we used the internal consistency approach (Cronbach’s alpha) to assess the reliability of the scale. Kline and Walters (2016) suggested that the value of alpha with 0.7 or higher shows better reliability (see Table 2, for the corresponding values of variables of the study).

**TABLE 2:** Cronbach’s alpha, standard deviation, mean, and variance.

**Measurement scale**	**Number of items**	**Cronbach’s alpha**	**Min**	**Max**	**Mean**	**Std. deviation**	**Skewness**	**Kurtosis**
ICT use	4	0.874	1.00	5.00	3.9279	0.56880	−0.516	1.686
Innovation	11	0.932	2.00	5.00	4.3175	0.49584	−0.641	1.006
Absorptive capacity	11	0.935	3.00	5.00	4.3407	0.50075	−0.430	−0.392
Temporal leadership	7	0.932	1.00	5.00	3.5631	0.76434	−0.528	0.626
Corporate entrepreneurship	6	0.922	2.00	5.00	4.1455	0.52045	−0.420	1.248
Competitive advantage	7	0.836	2.00	5.00	3.7665	0.69555	0.138	1.435

Similarly, a bivariate correlation analysis was carried out to analyze the strength and direction of the relationships. The results shown in Table 3 indicate positive and significant correlations among ICT use, innovation, absorptive capacity, temporal leadership, corporate entrepreneurship, and competitive advantage.

**TABLE 3:** Correlations among the constructs.

**Constructs**	**IN**	**CE**	**AC**	**TL**	**CA**	**ICT**
Innovation	1	0.304**	0.352**	0.074	0.202**	0.082
Corporate entrepreneurship	0.304**	1	0.280**	0.347**	0.347**	0.359**
Absorptive capacity	0.352**	0.280**	1	0.046	0.091	0.101*
Temporal leadership	0.074	0.347**	0.046	1	0.150**	0.318**
Competitive advantage	0.202**	0.347**	0.091	0.150**	1	0.308**
Information and communication technologies (ICT use)	0.082	0.359**	0.101*	0.318**	0.308**	1

*p < 0.5 and **p < 0.01.

### Measurement Model

In evaluating the measurement model, factor analysis is a statistical technique that can be employed to analyze constructs in terms of their underlying factors (Hair et al., 2010). In this research, goodness of fit of the measurement model was examined through CMIN (*χ*^2^), NFI, IFI, comparative fit index (CFI), GFI, and RMSEA. In order to achieve a model’s suitability, the value of relative CMIN must be less than 5.0 (Bentler and Chou, 1987), and the value for our model is CMIN/*df* = 1.845, suggesting an acceptable fit for the model. The RMSEA value should be less than 0.08 for the data to be adopted (Schumacker and Lomax, 2004). Fortunately, this fit measure with the value of 0.047 also demonstrates goodness of fit of the model to the data. The model fitness has also been established with other indicators complying the threshold values as shown in Table 4.

**TABLE 4:** Model fit indicators.

**Measure**	**Estimate**	**Threshold**	**Interpretation**
CMIN	1713.867	–	–
*df*	929.000	–	–
CMIN/*df*	1.845	Between 1 and 3	Excellent
CFI	0.934	>0.95	Acceptable
SRMR	0.043	<0.08	Excellent
RMSEA	0.047	<0.06	Excellent
PClose	0.924	>0.05	Excellent

**FIGURE 2:**
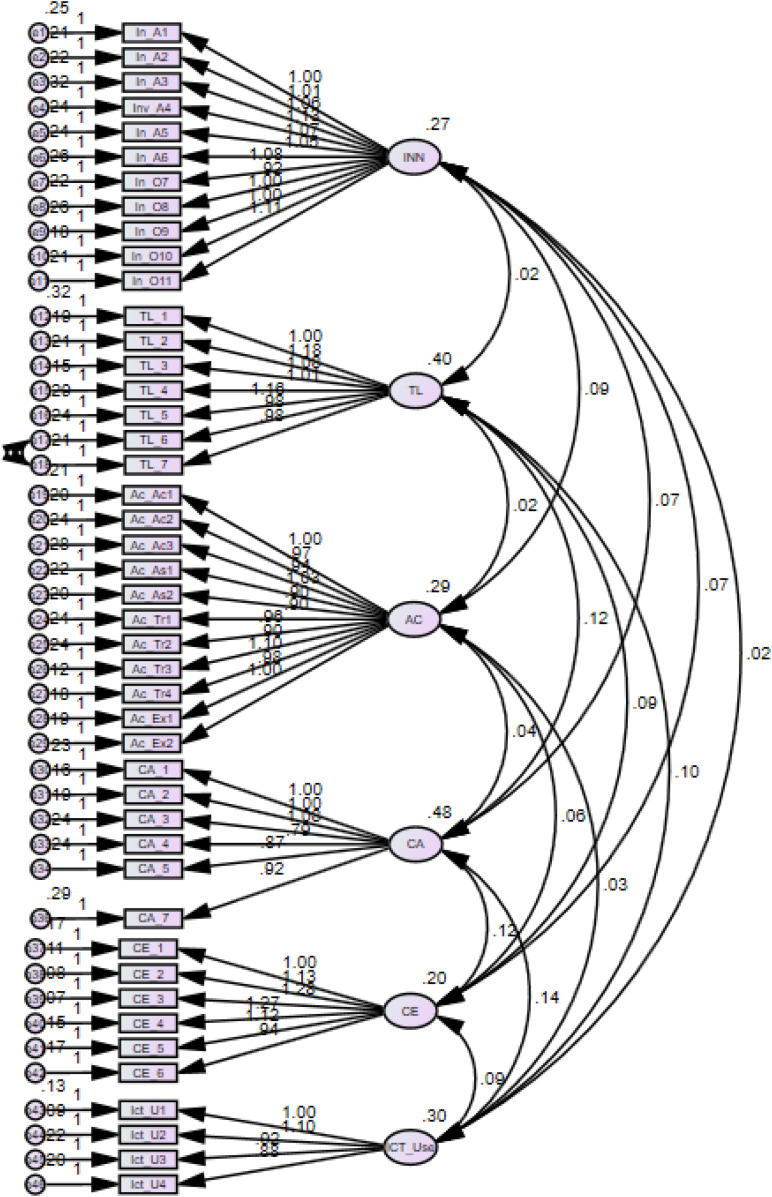
Confirmatory factor analysis.

Validity is another prerequisite for determining a measure’s goodness after reliability analysis. The construct validity has been established after conforming the convergent validity, discriminant validity, and face validity. These items were also measured and adopted in the past studies, so face validity was established. On the other hand, convergent validity was demonstrated by factor loadings and average variance of constructs extracted (AVE) with minimum criteria of cutoff as AVE > 0.5 (Al-Refaie, 2015). As shown in Figure 2, the CFA results indicate that all the items are significant with *p* < 0.001, and factor loadings are greater than 0.50. Similarly, all the constructs possess AVE value more than 0.5 (Table 5), thereby supporting the convergent validity. Discriminant validity, on the other hand, determines the magnitude of unique difference between measurements of different latent variables. It is measured by comparing the shared AVE of the square root of latent constructs’ respective interconstruct correlation estimates. It can be seen from Table 5 that square roots of AVE of all constructs in the diagonal are more than their corresponding interconstruct correlations. Therefore, the proposed measurement model exhibits discriminant validity.

**TABLE 5:** Average variance extracted (AVE) and discriminant validity.

	**AVE**	**ICT use**	**Innovation**	**AC**	**CE**	**CEOs’ TL**	**CA**
**ICT use**	0.637	**0.798**					
**Innovation**	0.556	0.064	**0.746**				
**AC**	0.570	0.107	0.317	**0.755**			
**CE**	0.665	0.379	0.320	0.248	**0.816**		
**CEOs’ TL**	0.660	0.301	0.070	0.050	0.341	**0.813**	
**CA**	0.650	0.363	0.203	0.120	0.378	0.280	**0.806**

*For all the constructs, square roots of AVE (in bold) are shown as diagonal elements and interconstruct correlations as off-diagonal.*

### Structural Model (Hypotheses Testing)

**FIGURE 3:**
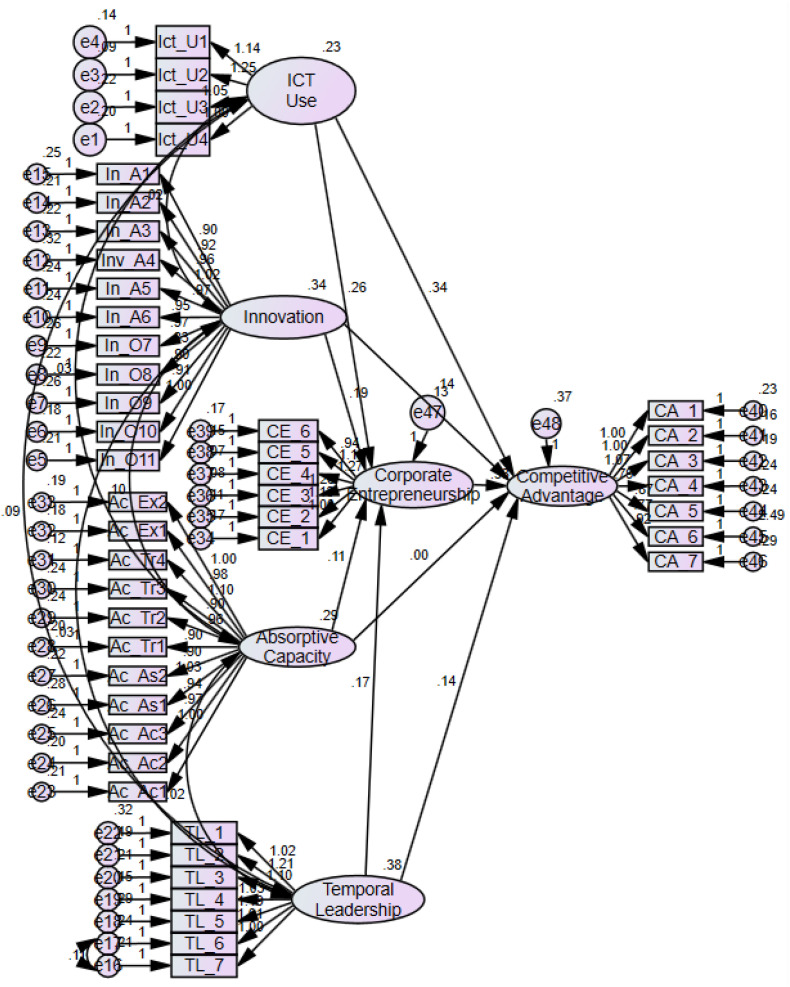
Regression estimates structural models.

The regression estimates conducted through AMOS 24, shown in Figure 3, are summarized in Table 6. The overall fit measures for the regression model demonstrate that the model fits the data well (CMIN/*df* = 1.845, NFI = 0.867, TLI = 0.929, CFI = 0.934, GFI = 0.835, and RMSEA = 0.047).

**TABLE 6:** Direct effects of the structural model.

**Hypothesis**	**Path**			**β**	***SE***	**CR**	***p***	**Result**
H_1_	CE	←	ICT use	0.259	0.051	5.121	***	Supported
H_2_	CE	←	INN	0.187	0.04	4.677	***	Supported
H3	CE	←	AC	0.105	0.041	2.55	0.011	Supported
H4	CE	←	TL	0.168	0.037	4.497	***	Supported
H_5_	CA	←	CE	0.326	0.096	3.404	***	Supported
H_6_	CA	←	ICT use	0.345	0.085	4.074	***	Supported
H_7_	CA	←	INN	0.133	0.066	2.028	0.043	Supported
H_8_	CA	←	AC	−0.003	0.068	−0.042	0.967	Not supported
H_9_	CA	←	TL	0.144	0.062	2.338	0.019	Supported

***p < 0.001.

The results in Table 6 for hypothesis H_1_ corroborate that information and communication technologies is positively related to corporate entrepreneurship. The values of the estimates are 0.259, the standard error is 0.051, *p*-value is significant at 0.000 level, while the critical ratio of ICT use on corporate entrepreneurship is 5.121. The results for H_2_ (the estimate is 0.187, standard error is 0.040, *p*-value is significant at 0.000, and critical ratio is 4.667) demonstrate that innovation has a significant and positive impact on corporate entrepreneurship. The findings for hypothesis H_3_ (estimate = 0.105, standard error = 0.041, critical ratio = 2.550, and *p*-value = 0.011) reveal that absorptive capacity significantly affects corporate entrepreneurship. In our analysis (estimate = 0.168, *p*-value = 0.000, standard error = 0.037, and critical ratio = 4.497), the results for hypothesis H_3_ establish that temporal leadership has also a significant relation with corporate entrepreneurship.

The regression analysis has also computed estimated direct effects of predictors on competitive advantage. The estimated regression weight of ICT use (estimate = 0.345, *p*-value = 0.000, *SE* = 0.085, and CR = 4.074) on competitive advantage (H_6_) shows that ICT use significantly influences competitive advantage. Similarly, innovation has shown a significant positive impact on competitive advantage (H_7_) (estimate = 0.133, *p*-value = 0.043, *SE* = 0.066, and critical ratio = 2.028). The value of the direct effect of absorptive for hypothesis H_8_ on competitive advantage is −0.003 and the *p*-value is insignificant (0.967), which indicates an insignificant relationship between absorptive capacity and competitive advantage. The results also suggest a significant relationship between temporal leadership and competitive advantage—hypothesis H_9_ (estimate = 0.144, *p*-value = 0.019, and critical ratio = 2.338). Finally, the result for hypothesis H_5_ also confirms that corporate entrepreneurship has a significant direct impact on competitive advantage (estimates = 0.326, standard error = 0.096, *p*-value = 0.000, and critical ratio = 3.404). With reference to H_10_, there is an indirect effect of ICT use, innovation, absorptive capacity, and temporal leadership on competitive advantage through corporate entrepreneurship as a mediator. The significance values of the indirect relationships were determined in AMOS through bootstrapping procedure based on 2,000 bootstrap samples. The direct effects of ICT use, innovation, absorptive capacity, and temporal leadership on competitive advantage are 0.345, 0.133, −0.003, and 0.144, respectively, with *p*-values less than 0.05 except for the relationship of absorptive capacity. On the other hand, the corresponding indirect effects of ICT use, innovation, absorptive capacity, and temporal leadership on competitive advantage are 0.084, 0.061, 0.034, and 0.055 with *p*-values less than 0.05 showing that corporate entrepreneurship mediates all the relationships as reported in Table 7. In contrast, the strengths of mediating effects were determined by computing variance accounted for (VAF) value. The VAF value greater than 80% is considered to be full mediation, the value from 20 to 80% indicates partial mediation, while there was no mediation if the value is less than 20% (Hair et al., 2013). It can be observed from Table 7 that all mediation effects are of medium level (VAF ≥ 20%), except for AC that has full mediation (VAF > 80%).

**TABLE 7:** Indirect effects (mediation) obtained through bootstrapping.

**Hypothesis**	**Path**			**β**	**VAF = effects (indirect/total) %**	***p***	**Result**
H_10–A_	CA	←	CE ← ICT use	0.084	20%	0.005	Partial mediation
H_10–B_	CA	←	CE ← INN	0.061	31%	0.002	Partial mediation
H_10–C_	CA	←	CE ← AC	0.034	92%	0.003	Full mediation
H_10–D_	CA	←	CE ← TL	0.055	28%	0.002	Partial mediation

## Discussion

The proposed research model corroborates and expands the literature to correlate ICT use, innovation, absorptive capacity and CEO’s temporal leadership with corporate entrepreneurship, and finally, with competitive advantage. Although the converse relationships are also possible (Bollen and Pearl, 2013), the discussion is limited to only one-sided relationships due to stipulated frame of work. The outcomes of H_1_ through H_4_ establish the relevance of predictors as enablers of corporate entrepreneurship. These remarkable effects suggest that by investing in ICT use, innovation, absorptive capacity, and CEO’s temporal leadership, IT firms can own ingredients of good corporate entrepreneurship. The results established here are in agreement with previous studies. For example, the results of H_1_ (β = 0.259, *p*-value < 0.001) are supported by Hosseini et al. (2014) and Tang et al. (2015), who concluded that the relationship between ICT and corporate entrepreneurship is significant. Similarly, innovation and corporate entrepreneurship (H_2_) show a positive and significant relationship (β = 0.187, *p*-value < 0.001). This result is corroborated by the findings of Martín-Rojas et al. (2017). On the other hand, the results supporting H_3_ (β = 0.105, *p*-value = 0.011) reveal that absorptive capacity is significantly and positively related to corporate entrepreneurship. The finding is similar to the conclusion made by García-Sánchez et al. (2018). Likewise, similar to the results for H_4_ (β = 0.168, *p*-value < 0.001), Maruping et al. (2015) have proposed the identical findings substantiating the relationship between CEO’s temporal leadership and corporate entrepreneurship. These relationships then converge on the competitive advantage through corporate entrepreneurship (H_5_). The empirical positive results (β = 0.326, *p*-value < 0.000) are ratified by the findings of previous research work (Daryani and Karimi, 2017; Prange and Pinho, 2017). It validates that effective employment of corporate entrepreneurship contributes toward fostering competitive advantage. It is organizational capability and situation used as a resource to achieve competitive advantage (Stevens et al., 2015). It is also an activity of firm’s capabilities to achieve the desired result (Stevens et al., 2015).

Furthermore, the direct effects of the predictors, i.e., ICT use, innovation, absorptive capacity, and CEO’s temporal leadership on competitive advantage as the outcome, have been explained under hypotheses H_6_, H_7_, H_8_, and H_9_, respectively. The corresponding results (β = 0.345, *p*-value < 0.000; β= 0.133, *p*-value = 0.045; β = −0.003, *p*-value = 0.967; β = 0.144, *p*-value = 0.019) are consistent with the relevant previous studies. The results suggest that all these variables show positive and significant impact on competitive advantage, except absorptive capacity that does not impact the competitive advantage directly. Managing resources of ICT for enhancing the firm performance and attaining competitive advantage requires a firm culture, which may help in identifying, making, and assessing these opportunities (Agarwal and Brem, 2015). The effective use of ICT contributes toward the successful organization and competitive advantage (Manochehri et al., 2012). In the same way, innovation can transform information and communication technology resources, the firm’s practices, and explicit and tacit knowledge into beneficial capabilities, initiatives, and resources; therefore, competitive advantage can be achieved through innovation (Agarwal and Brem, 2015). In addition, absorptive capacity helps attain competitive advantage through exploitation and exploration that enhances market share, sale of firm, and profitability than the other companies (Martín-Rojas et al., 2013). On the contrary, our results suggest that this effect is exploitable through corporate entrepreneurship instead of its direct applicability.

On the other hand, all the indirect effects are significant and positive, as reported in Table 7. Encapsulating, based upon the empirical evidences, corporate entrepreneurship partially mediates the relationships of ICT use (H_10–A_), innovation (H_10–B_), and CEO’s temporal leadership (H_10–C_) with competitive advantage to the extent of 20, 31, and 28%, respectively. On the other hand, CE fully mediates between absorptive capacity and competitive advantage (H_10–D_) achieving the magnitude of 92%. It implies that absorptive capacity can be exploited to achieve competitive advantage meaningfully only through venturing entrepreneurship at the corporate level. Thus, we confirm that ICT, innovation, absorptive capacity, and temporal leadership coupled with corporate entrepreneurship help develop entrepreneurial activities, and their upshots attain more competitive advantage and ambitious goals.

## Managerial Implications

In this study, we emphasize the pivotal role played by strategic resources such as information and communication technologies, innovation, absorptive capacity, temporal leadership, and corporate entrepreneurship to better seize the opportunities in enhancing the firms’ competitiveness. At the practical level, this study has implications for managers, entrepreneurs, innovation adopters, and technology suppliers to better understand and transform dynamic capabilities and value creation resources into competitive advantage, which may further help them face global challenges. Our results have significance for policy makers as well, who may formulate policies that foster a culture of corporate entrepreneurship, and provide facilitating conditions such as entrepreneurial education, training, and an enabling environment to better exploit the opportunities offered by ICT, absorptive capacity, temporal leadership, and innovation. It may also enable firms to identify their strengths and weaknesses for increasing long-term competitiveness and profitability. Firms that have absorptive capacity can interact with the external environment innovatively to access external knowledge necessary for generating new product ideas to help attain additional competitive advantage. Consequently, this would produce a ripple effect in the form of jobs creation, greater exports, reduced imports, and growth in national GDP. In a nutshell, we suggest that corporate entrepreneurship channelizes the parameters required to reap the advantages of opportunities for an organization.

On the social front, this study can improve the quality of life of the poor by offering people with equal opportunities who face difficulties. Organizations can foster their personnel’s entrepreneurial skills through trainings, workshops, mentoring, and motivation to further strengthen the organization. By adopting corporate entrepreneurship, managers can increase job opportunities by creating new markets with its impact on human resources and long-term competitive advantage. The growth and exports of any country considerably depend on companies’ competitiveness that are operating in the country. Consequently, countries can benefit from the competitiveness and innovative activities of the companies by implementing polices that incentivize them.

## Conclusion

Today’s information age and globalized environment reveal contemporary challenges that cannot be underrated. Despite these challenges, many opportunities can be gained through the proper use of resources for sustaining competitive advantage. ICT, innovation, absorptive capacity, and temporal leadership are strategic resources of an organization that play their role to achieve competitive advantage. However, these potential benefits can be realized in an environment fostered by entrepreneurial spirit. This paper asserts that ICT, absorptive capacity, temporal leadership, and innovation have a positive impact on competitiveness of an organization if opportunities are managed within the culture of an organization through corporate entrepreneurship. In this way, firms not only can maintain their present competitive advantage but also can cope with challenges and threats by exploiting new opportunities. The results are established on SEM analysis conducted on data collected from 460 IT firms through attitudinal measures of scale. According to the regression results, ICT use, innovation, absorptive capacity, and CEO’s temporal leadership show significant direct and indirect impacts on competitive advantage through corporate entrepreneurship. These results are consistent with past studies conducted by various researchers [e.g., Manochehri et al. (2012); Hosseini et al. (2014), Agarwal and Brem (2015); Tang et al. (2015)].

The study has several implications for managers, entrepreneurs, innovation adopters, and technology suppliers to better understand and transform dynamic capabilities and value creation resources into competitive advantage, which may further help them face global challenges. Managers can make use of these significant elements to help maintain competitive advantage and for the creation of wealth, increase in sales, and growth of market share. It also has significance for policy makers, who may formulate policies that foster a culture of corporate entrepreneurship and provide facilitating conditions such as entrepreneurial education, training, and an enabling environment to better exploit the opportunities offered by ICT, absorptive capacity temporal leadership, and innovation. It may also assist firms to identify their strengths and weaknesses for increasing long-term competitiveness and profitability. Firms that have absorptive capacity can interact with the external environment innovatively to access external knowledge necessary for generating new product ideas to help attain additional competitive advantage. Consequently, this would produce a ripple effect in the form of jobs creation, greater exports, reduced imports, and growth in national GDP.

In spite of several contributions, the present study has some limitations that may turn out to be an opportunity for further research. First, the data were collected through convenient sampling in one district of Lahore, which limits the generalizability of the results. The prospective research should be performed using a more representative probabilistic sampling technique and collecting data from other IT hubs of the country. Likewise, the proposed model or its adaptation may be cross-checked for its reliability in other entrepreneurial sectors. Moreover, granted that common method bias was not a perceptible issue, we still emphasize on applying alternative solutions to address this concern. Another point for the prospective work is to test endogeneity to detect endogenous regressors through the Hausman test.

## Data Availability Statement

The datasets generated for this study are available on request to the corresponding author.

## Ethics Statement

Ethical review and approval was not required for the study on human participants in accordance with the local legislation and institutional requirements. The patients/participants provided their written informed consent to participate in this study.

## Author Contributions

All authors listed have made a substantial, direct and intellectual contribution to the work, and approved it for publication.

## Conflict of Interest

The authors declare that the research was conducted in the absence of any commercial or financial relationships that could be construed as a potential conflict of interest.
