# Supplementary material for: Ahead of the Curve: Leveraging Antecedents of Corporate Entrepreneurship to Pull Off Competitive Advantage
Source: Front Psychol. 2020 Oct 26;11:531886. doi: 10.3389/fpsyg.2020.531886 (PMC7649392; doi:10.3389/fpsyg.2020.531886)
Supplement: Supplementary file 1 [file Data_Sheet_1.docx]

# Appendix

| **The following section covers the questions on your views about the Information and Communication Technologies (ICT USE). Record your responses on 1 – 5 scale given below:** | | | | | | | | | | | |  |
| --- | --- | --- | --- | --- | --- | --- | --- | --- | --- | --- | --- | --- |
| **Strongly disagree** | | **Disagree** | **Neutral** | **Agree** | | **Strongly Agree** | | | | | | |
| **1** | | **2** | **3** | **4** | | **5** | | | | | | |
| 1 | a. Our organization is frequent user of organization's information and communication technologies and Information System. | | | |  | | 1 | 2 | 3 | 4 | 5 | |
| 2 | b. I Consider myself a frequent user of my organizations information technology and systems. | | | |  | | 1 | 2 | 3 | 4 | 5 | |
| 3 | c. Our information and communication technologies integration in work processes. | | | |  | | 1 | 2 | 3 | 4 | 5 | |
| 4 | d. We use information and communication technologies and information system capabilities. | | | |  | | 1 | 2 | 3 | 4 | 5 | |

| **The following section covers the questions on your views about the Innovation (ATM & OPR**)**. Record your responses on 1 – 5 scale given below:** | | | | | | | | | | | |  |
| --- | --- | --- | --- | --- | --- | --- | --- | --- | --- | --- | --- | --- |
| **Strongly disagree** | | **Disagree** | **Neutral** | **Agree** | | **Strongly Agree** | | | | | | |
| **1** | | **2** | **3** | **4** | | **5** | | | | | | |
|  | **Innovation Atmosphere (INVATM**) | | | |  | |  |  |  |  |  | |
| 5 | a. Our organization is pursuit of novel knowledge. | | | |  | | 1 | 2 | 3 | 4 | 5 | |
| 6 | b. Our organization searches for latest technology. | | | |  | | 1 | 2 | 3 | 4 | 5 | |
| 7 | c. Investigation in various directions. | | | |  | | 1 | 2 | 3 | 4 | 5 | |
| 8 | d. Our organization explore new areas. | | | |  | | 1 | 2 | 3 | 4 | 5 | |
| 9 | e. Our organization discover new products. | | | |  | | 1 | 2 | 3 | 4 | 5 | |
| 10 | f. Our organization has breakthrough improvements in products/profits. | | | |  | | 1 | 2 | 3 | 4 | 5 | |
|  | **Innovation opportunities (INVOPR)** | | | |  | |  |  |  |  |  | |
| 11 | a. Opportunities for product innovation are abundant in our industry. | | | |  | | 1 | 2 | 3 | 4 | 5 | |
| 12 | b. Opportunities for technological innovation are abundant in our industry. | | | |  | | 1 | 2 | 3 | 4 | 5 | |
| 13 | c. We are spending High on Research & Development in industry. | | | |  | | 1 | 2 | 3 | 4 | 5 | |
| 14 | d. We are spending High on Research & Development in company. | | | |  | | 1 | 2 | 3 | 4 | 5 | |
| 15 | e. Our products/services require the adoption of new and different methods and procedures. | | | |  | | 1 | 2 | 3 | 4 | 5 | |

| **The following section covers the questions on your views about the Absorptive Capacity. Record your responses on 1 – 5 scale given below:** | | | | | | | | | | | |  |
| --- | --- | --- | --- | --- | --- | --- | --- | --- | --- | --- | --- | --- |
| **Strongly disagree** | | **Disagree** | **Neutral** | **Agree** | | **Strongly Agree** | | | | | | |
| **1** | | **2** | **3** | **4** | | **5** | | | | | | |
|  | **Absca1 (Knowledge Acquisition)** | | | |  | |  |  |  |  |  | |
| 16 | a. There is Close personal interaction between the two organizations. | | | |  | | 1 | 2 | 3 | 4 | 5 | |
| 17 | b. The relationship between the two organizations is characterized by mutual trust. | | | |  | | 1 | 2 | 3 | 4 | 5 | |
| 18 | c. The relationship between the two organizations is characterized by a high level of reciprocity. | | | |  | | 1 | 2 | 3 | 4 | 5 | |
|  | **Absca2 (Knowledge Assimilation)** | | | |  | |  |  |  |  |  | |
| 19 | a. The organizational cultures of the two organizations are compatible. | | | |  | | 1 | 2 | 3 | 4 | 5 | |
| 20 | b. The operating and management styles of the two organizations are compatible. | | | |  | | 1 | 2 | 3 | 4 | 5 | |
|  | **Absca3 (Knowledge Transformation)** | | | |  | |  |  |  |  |  | |
| 21 | a. Interdepartmental meetings are organized to discuss the development and tendencies of the organization. | | | |  | | 1 | 2 | 3 | 4 | 5 | |
| 22 | b. The important data are transmitted regularly to all units. | | | |  | | 1 | 2 | 3 | 4 | 5 | |
| 23 | c. When something important occurs, all units are informed within a short time. | | | |  | | 1 | 2 | 3 | 4 | 5 | |
| 24 | d. The organization has the capabilities or abilities necessary to ensure that knowledge flows within the organization and is shared among the different units. | | | |  | | 1 | 2 | 3 | 4 | 5 | |
|  | **Absca4 (Knowledge Exploitation)** | | | |  | |  |  |  |  |  | |
| 25 | a. The division of functions and responsibilities regarding use of information and knowledge obtained from outside the organization is clear. | | | |  | | 1 | 2 | 3 | 4 | 5 | |
| 26 | b. The organization has the capabilities and abilities needed to exploit the information and knowledge obtained from outside the organization. | | | |  | | 1 | 2 | 3 | 4 | 5 | |

| **The following section covers the questions on your views about the Corporate Entrepreneurship. Record your responses on 1 – 5 scale given below:** | | | | | | | | | | | |  |
| --- | --- | --- | --- | --- | --- | --- | --- | --- | --- | --- | --- | --- |
| **Strongly disagree** | | **Disagree** | **Neutral** | **Agree** | | **Strongly Agree** | | | | | | |
| **1** | | **2** | **3** | **4** | | **5** | | | | | | |
| 27 | a. There are dramatic changes in our products and service mix over the past three years. | | | |  | | 1 | 2 | 3 | 4 | 5 | |
| 28 | b. We are emphasizing on major innovations in products and services over the past three years. | | | |  | | 1 | 2 | 3 | 4 | 5 | |
| 29 | c. Tendency for high risk projects over the past three years. | | | |  | | 1 | 2 | 3 | 4 | 5 | |
| 30 | d. Introduced new products and services over the past three years OR This company has emphasized taking bold, wide-ranging action in positioning itself and its product (services) over the past three years. | | | |  | | 1 | 2 | 3 | 4 | 5 | |
| 31 | e. Strong commitment to research and development (R&D), technological leadership, and innovation. | | | |  | | 1 | 2 | 3 | 4 | 5 | |
| 32 | f. Followed strategies that allow it to exploit opportunities in its external environment. | | | |  | | 1 | 2 | 3 | 4 | 5 | |

| **The following section covers the questions on your views about the competitive advantage. Record your responses on 1 – 5 scale given below:** | | | | | | | | | | | | | | | | | | | | | | |  |
| --- | --- | --- | --- | --- | --- | --- | --- | --- | --- | --- | --- | --- | --- | --- | --- | --- | --- | --- | --- | --- | --- | --- | --- |
| **Strongly disagree** | | | | **Disagree** | | **Neutral** | | **Agree** | | | | **Strongly Agree** | | | | | | | | | | | |
| **1** | | | | **2** | | **3** | | **4** | | | | **5** | | | | | | | | | | | |
| 33 | | a. Our organization performance is better than rivals. | | | | | | | |  | | | | 1 | | 2 | | 3 | | 4 | | 5 | |
| 34 | | b. We have high efficiency levels in operations. | | | | | | | |  | | | | 1 | | 2 | | 3 | | 4 | | 5 | |
| 35 | | c. Our organization Productivity is high. | | | | | | | |  | | | | 1 | | 2 | | 3 | | 4 | | 5 | |
| 36 | | d. Our Organization market constantly growing. | | | | | | | |  | | | | 1 | | 2 | | 3 | | 4 | | 5 | |
| 37 | | e. The employee satisfaction level is high in our organization. | | | | | | | |  | | | | 1 | | 2 | | 3 | | 4 | | 5 | |
| 38 | | f. Our customers are satisfied. | | | | | | | |  | | | | 1 | | 2 | | 3 | | 4 | | 5 | |
| 39 | | g. Overall, company performance is high and improving. | | | | | | | |  | | | | 1 | | 2 | | 3 | | 4 | | 5 | |
| **The following section covers the questions on your views about the Temporal leadership. Record your responses on 1 – 5 scale given below:** | | | | | | | | | | | | | | | | | | | | | | |  |
| **Strongly disagree** | | | **Disagree** | | **Neutral** | | **Agree** | | | | **Strongly Agree** | | | | | | | | | | | | |
| **1** | | | **2** | | **3** | | **4** | | | | **5** | | | | | | | | | | | | |
| 40 | a. Your project leader reminds members of important deadlines. | | | | | | | |  | | | | 1 | | 2 | | 3 | | 4 | | 5 | | |
| 41 | b. Your project leader prioritizes tasks and allocate time to each task. | | | | | | | |  | | | | 1 | | 2 | | 3 | | 4 | | 5 | | |
| 42 | c. Your project leader prepares and build in time for contingencies, problems, and emerging issues. | | | | | | | |  | | | | 1 | | 2 | | 3 | | 4 | | 5 | | |
| 43 | d. Your project leader pace the team so that work is finished on time. | | | | | | | |  | | | | 1 | | 2 | | 3 | | 4 | | 5 | | |
| 44 | e. Your project leader urge members to finish subtasks on time. | | | | | | | |  | | | | 1 | | 2 | | 3 | | 4 | | 5 | | |
| 45 | f. Your project leader set milestones to measure progress on the project. | | | | | | | |  | | | | 1 | | 2 | | 3 | | 4 | | 5 | | |
| 46 | g. Your project leader effective in coordinating the team to meet client deadlines. | | | | | | | |  | | | | 1 | | 2 | | 3 | | 4 | | 5 | | |
